# The presurgical controlling nutritional status (CONUT) score is independently associated with severe peristomal skin disorders: a single-center retrospective cohort study

**DOI:** 10.1038/s41598-021-98369-y

**Published:** 2021-09-22

**Authors:** Takuya Shiraishi, Hiroomi Ogawa, Chika Katayama, Katsuya Osone, Takuhisa Okada, Ryuji Katoh, Akihiko Sano, Makoto Sakai, Makoto Sohda, Ken Shirabe, Hiroshi Saeki

**Affiliations:** 1 Division of Gastroenterological Surgery, Department of General Surgical Science, Gunma University Graduate School of Medicine, 3-39-22 Showa-machi, Maebashi, Gunma 371-8511 Japan; 2 Department of General Surgical Science, Gunma University Graduate School of Medicine, 3-39-22 Showa-machi, Maebashi, Gunma 371-8511 Japan

**Keywords:** Diseases, Gastroenterology, Medical research, Risk factors

## Abstract

While nutritional interventions may potentially lower the risk of peristomal skin disorders (PSDs) and their exacerbation, no previous studies have evaluated the relationship between PSDs and nutritional status using the Controlling Nutritional Status (CONUT) score. The purpose of this study was to assess the impact of preoperative nutritional status on stoma health, and determine risk factors for postoperative PSDs, including severe PSDs. A retrospective analysis was performed of 116 consecutive patients with rectal cancer who underwent radical surgery with ileostomy or colostomy creation. PSDs were diagnosed in 32 patients (27.6%); including 10 cases (8.7%) that were defined as severe based on the ABCD-stoma score. Multivariable logistic regression showed that smoking (odds ratio [OR] 3.451, 95% confidence interval [CI] 1.240–9.607, p = 0.018) and ileostomy (OR 3.287, 95% CI 1.278–8.458, p = 0.014) were independent risk factors for PSDs. A separate multivariable logistic regression analysis of risk factors for severe PSDs, found that the only independent risk factor was the CONUT score (OR 10.040, 95% CI 1.191–84.651, p = 0.034). Severe PSDs are associated with preoperative nutritional disorders, as determined by the CONUT score. Furthermore, nutritional disorders may increase the severity of PSDs, regardless of the stoma type.

## Introduction

Following the creation of a stoma, up to 80% of patients experience stoma-related complications, which predominantly consist of peristomal skin disorders (PSDs). While the majority are not severe, PSDs commonly cause itching and pain, which lower patient quality of life. They also necessitate the frequent replacement of the stoma appliance, which increases the financial burden on the patient^1–4^. While standardized best practice guidelines (e.g., preoperative stoma site marking, ongoing involvement of a stoma care nurse, correct use of stoma appliances) are used to prevent PSDs, the incidence of this postoperative complications remains high^3,5^. Therefore, there is a need to develop further interventions to reduce or eliminate PSD occurrence.

Nutritional deficiencies are related to skin disorders. High metabolic turnover of body parts such as skin, hair, and nails requires a good supply of nutrients and energy^6^. Patients with nutrient deficiencies have delayed wound healing and are more likely to develop wound complications, because nutrient deficiencies can adversely affect the growth of skin tissue form and wound healing^7^. The skin around the stoma is likely to be exposed to stimuli such as digestive fluid and feces. Therefore, patients with nutritional disorders are at high risk of developing PSDs, and nutritional management before and after surgery with stoma construction including ileostomy and colostomy may be very important.

The prognostic nutritional index (PNI), geriatric nutritional risk index (GNRI), and controlling Nutritional Status (CONUT) are used clinically as indicators of nutritional disorders. These indexes have been reported to be a valuable objective and comprehensive tool for assessing a patient’s general nutritional status. The PNI is calculated based on the serum albumin concentration and total lymphocyte count, which reflects both the nutritional and immune status of the patient^8^. Many studies have reported that PNI was associated with postoperative complications and survival in patients with various cancers^9^. The GNRI is calculated based on height, weight, and serum albumin levels. The GNRI has been used as a nutrition-related risk indicator of morbidity and mortality for older patients, and is a geriatric-specific index^10^. The CONUT score is calculated based on serum albumin, total cholesterol concentration, and total lymphocyte count, which reflect protein metabolism, lipid metabolism, and immune function, respectively^11^. The validity and cost-effectiveness of the CONUT score have been reported previously^12,13^. However, the utility of this index has not been assessed in patients with stomas, and no previous studies have evaluated its relationship with PSD severity.

The purpose of this study was to evaluate the impact of preoperative nutritional status, as assessed by the CONUT score, prognostic nutritional index (PNI), and geriatric nutritional risk index (GNRI), on stoma health. Furthermore, we aimed to determine risk factors for postoperative PSDs and severe PSDs.

## Methods

### Patient data collection

This retrospective study included all consecutive patients who underwent rectal cancer surgery with stoma creation, including ileostomy and colostomy, from June 2013 to March 2020 at the Gunma University Hospital in Japan. All patients with primary rectal cancer were identified. Exclusion criteria were as follows: (1) patients who did not undergo radical surgery, (2) patients with missing values in the scoring systems, and (3) patients who were not followed up after surgery due to transfer to another facility. Patient characteristics (sex, age, body mass index [BMI], smoking, hypertension [HT], diabetes mellitus [DM], history of preoperative chemoradiotherapy, preoperative nutritional status, clinical TNM classification, final TNM classification, and use of adjuvant chemotherapy) and surgical characteristics (operation type, approach type [laparoscopy or open], operation time, blood loss, stoma type [ileostomy or colostomy], temporary stoma, and stoma position [upper or lower abdomen]) were extracted from medical and surgical reports. The condition of the stoma was observed during hospitalization by surgeons and nurses (including wound, ostomy, and continence [WOC] nurses). Thereafter, the patients were assessed during follow-up outpatient visits. The study protocol was approved by the Institutional Review Board of Gunma University Hospital (approval no. HS2020-196). The requirement for informed consent was waived because the analysis was based on the retrospective record review.

### Diagnosis of PSDs

**Figure 1:**
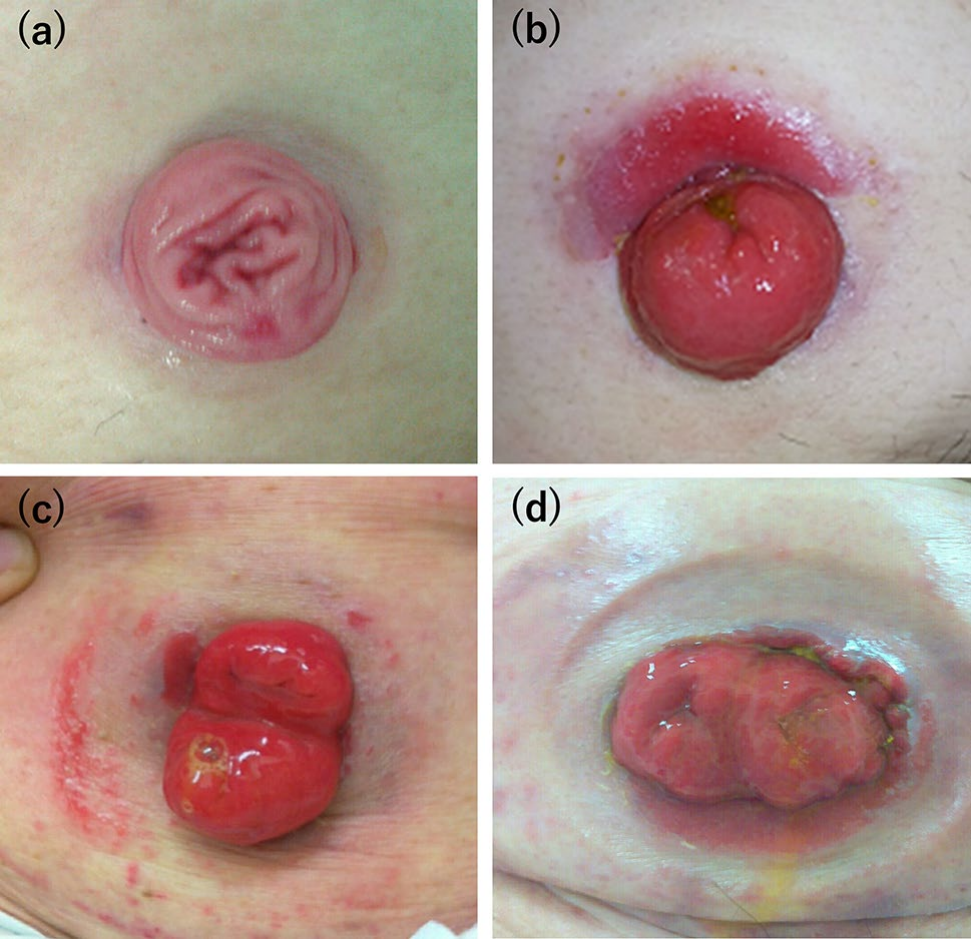
Peristomal skin disorders diagnosed according to the ABCD-stoma score. (**a**) Normal skin around stoma (score: 0, non-peristomal skin disorder). (**b**) Erosion on the upper side of the area near the stoma, where the skin barrier has dissolved (score: 2, non-severe peristomal skin disorder). (**c**) Erosion on the right side of the area near the stoma, where the skin barrier has dissolved. Erosion is observed in the region contacting the ostomy appliance (score: 4, severe peristomal skin disorder). (**d**) Ulcer and erosion on the area near the stoma, where the skin barrier has dissolved. Erythema is observed in the region contacting the ostomy appliance (score: 16, severe peristomal skin disorder).

The ABCD-stoma score was used for the diagnosis of PSDs and the assessment of their severity. This score was determined by evaluating changes in the skin, including erythema, erosion, blisters, pustules, ulcers, tissue overgrowth, and pigmentation (Fig. 1). The ABCD-stoma score was developed and published by the Japanese Society of Wound, Ostomy, and Continence Management in 2012^5^. The scoring scale evaluates the peristomal skin in three areas close to the stoma: adjacent (A), barrier (B), and circumscribing (C). It also evaluates discoloration (D). PSD severity was evaluated by medical staff as erythema 1, erosion 2, blister/pustule 3, or ulcer/tissue overgrowth 15 in the three skin areas. A score of 4 or higher, which requires a treatment period of > 28 days, was defined as severe PSD^5^.

PSDs and the score were evaluated by experienced WOC nurses and doctors who were blinded to the patients’ clinical history or outcomes. PSDs that occurred within 30 days after surgery were investigated because early PSDs may be related to preoperative nutrition. Severe PSDs that developed more than 30 days after surgery were only included if their initial onset was within 30 days after surgery.

### Evaluation of preoperative nutritional status

Preoperative nutritional status was assessed by the CONUT, PNI, and GNRI. The CONUT score was determined by measuring serum albumin and total cholesterol levels, as well as the total lymphocyte count, as described previously^11^. The scores and cutoff values for the serum albumin levels were 0 for ≥ 3.5 g/dL, 2 for 3.0–3.49 g/dL, 4 for 2.5–2.9 g/dL, and 6 for < 2.5 g/dL. The scores and cutoffs for the total cholesterol level were 0 for ≥ 180 mg/dL, 1 for 140–180 mg/dL, 2 for 100–139 mg/dL, and 3 for < 100 mg/dL. The scores and cutoffs for the total lymphocyte count were 0 for ≥ 1600 cells/µL, 1 for 1200–1599 cells/µL, 2 for 800–1199 cells/µL, and 3 for < 800 cells/µL. The sum of these scores was used as the CONUT score, and patients were divided into four categories as follows: normal (score 0–1), light (score 2–4), moderate (score 5–8), and severe (score 9–12). Classification as light, moderate, and severe indicated the presence of a nutritional disorder. PNI was calculated by inputting the serum albumin level and total lymphocyte count into the following formula^8^:

1 $$10\times serum \, albumin \, value \, (g/dL) + 0.005 \times peripheral \, lymphocyte \, count \, (/\mu L)$$

If the total yielded by Eq. (1) was less than 40, a PNI score of 1 was assigned; indicating the presence of a severe nutritional disorder, while a PNI score of 0 reflected a normal preoperative nutritional status. GNRI was calculated by inputting the serum albumin level and both the actual and ideal body weights into the following formula^10^:

2 $$1.489 \times albumin \, (g/L) + 41.7 \times (actual \, body \, weight/ideal \, body \, weight)$$

If the total yielded by Eq. (2) was > 98, the nutritional status was defined as normal. Blood samples from all patients were obtained within 1 month prior to surgery.

### Statistical analysis

Categorical variables were reported as the frequency and percentage of patients. Quantitative variables are reported as the median and range. Univariate and multivariable analyses of patient and surgical characteristics were conducted to compare cases with and without PSDs, as well as non-severe PSDs with severe PSDs (based on the ABCD-stoma score). Sex, age, BMI, smoking, HT, DM, chemoradiotherapy, clinical TNM classification, final TNM classification, albumin level, total cholesterol level, lymphocyte count, PNI, GNRI, CONUT, operation time, blood loss, approach type, stoma type, and stoma position were used as the independent factors for both comparisons. The multivariable analysis was performed using logistic regression with the backward stepwise method. Factors with p < 0.10 in the univariate analyses were included in separate multivariable analyses for all PSDs and severe PSDs. All statistical analyses were performed using SPSS (version 22.0; IBM Corp. Armonk, NY, USA), with the level of statistical significance set at p < 0.05.

### Ethics approval

This study was performed in line with the principles of the Declaration of Helsinki. Approval was granted by the Institutional Review Board of Gunma University Hospital (approval no. HS2020-196). Patients were not required to give informed consent to the study because the analysis used anonymous clinical data. And also, we applied Opt-out method to obtain consent on this study by using information disclosure document. The document was approved by the Institutional Review Board of Gunma University Hospital.

## Results

During the study period (June 2013–March 2020), we performed rectal cancer surgery with stoma creation on 160 patients. We excluded 23 patients who did not undergo radical surgery, 16 patients with missing values in the scoring systems, and 5 patients who were not followed up after surgery due to transfer to another facility. Finally, this study included 116 patients who underwent radical surgery with stoma creation for primary rectal cancer. Of the 116 patients, 83 were males (71.6%) and 33 were females (28.4%). The median age was 66 (39–88) years, and the median BMI was 22.0 (14.0–40.9) kg/m^2^, 72 patients (62.1%) were smokers, 39 patients (33.6%) had HT, 16 patients (13.8%) had DM, and 36 patients (31.0%) had received preoperative chemoradiotherapy. Laparoscopic and open approaches were performed in 85 (73.30%) and 31 (26.7%) patients, respectively. Stoma types comprised ileostomy in 63 patients (54.3%) and colostomy in 53 patients (45.7%). Upper and lower abdominal stomas were used in 21 (18.1%) and 95 (81.9%) patients, respectively. Patient characteristics are shown in Table 1.

**Table 1:** Characteristics of patients.

	N = 116
**Sex, N (%)**	
Male	83 (71.6)
Female	33 (28.4)
Age, median (range), years	66 (39–88)
Body mass index, median (range), kg/m^2^	22.0 (14.0–40.9)
**Smoking, N (%)**	
None	44 (37.9)
Yes	72 (62.1)
**Hypertension, N (%)**	
Absence	77 (66.4)
Presence	39 (33.6)
**Diabetes mellitus, N (%)**	
Absence	100 (86.2)
Presence	16 (13.8)
**Chemoradiotherapy, N (%)**	
Absence	80 (69.0)
Presence	36 (31.0)
**Clinical T status, N (%)**	
1	16 (13.9)
2	31 (26.7)
3	49 (42.2)
4	20 (17.2)
**Clinical N status, N (%)**	
0	73 (62.9)
1	37 (31.9)
2	6 (5.2)
**Clinical M status, N (%)**	
0	110 (94.8)
1	6 (5.2)
**Final T status, N (%)**	
1	15 (12.9)
2	39 (33.6)
3	41 (35.4)
4	21 (18.1)
**Final N status, N (%)**	
0	79 (68.2)
1	33 (28.4)
2	4 (3.4)
**Final M status, N (%)**	
0	111 (95.7)
1	5 (4.3)
Albumin level, median (range), g/dL	4.1 (1.8–5.0)
Total cholesterol level, median (range), mg/dL	193.0 (105.0–309.0)
Lymphocyte count, median (range), cells/uL	1.545.0 (440.0–3570.0)
**Operation type, N (%)**	
High anterior resection	4 (3.4)
Low anterior resection	51 (44.0)
Intersphincteric resection	14 (12.1)
Hartmann	7 (6.0)
Abdominal perineal resection	33 (28.4)
Total pelvic exenteration	6 (5.2)
Total colectomy	1 (0.9)
**Approach type, N (%)**	
Laparoscopy	85 (73.3)
Open	31 (26.7)
Operation time, median (range), mins	424.5 (146.0–809.0)
Blood loss, median (range), mL	195 (0–5507)
**Stoma type, N (%)**	
Colostomy	53 (45.7)
Ileostomy	63 (54.3)
Temporary stoma, N (%)	71 (61.2)
**Stoma position, N (%)**	
Upper abdomen	21 (18.1)
Lower abdomen	95 (81.9)
**Adjuvant chemotherapy, N (%)**	
Absence	80 (69.0)
Presence	36 (31.0)

PSDs were diagnosed in 32 patients (27.6%) a median of 12.0 (1.0–29.0) days post-surgery. Smoking (p = 0.011), and approach type (p = 0.040) and stoma type (p = 0.007) were significantly associated with PSDs in the univariate analysis (Table 2). Smoking (odds ratio [OR] 3.451, 95% confidence interval [CI] 1.240–9.607, p = 0.018) and ileostomy (OR 3.287, 95% CI 1.278–8.458, p = 0.014) were independent risk factors for PSDs in the multivariable logistic regression analysis.

**Table 2:** Risk factors for all peristomal skin disorders.

	No PSDs, N = 84	PSDs, N = 32	Univariate analysis		Multivariable analysis	
			OR (95% CI)	p	OR (95% CI)	p
**Sex, N (%)**						
Male	60 (71.4)	23 (71.9)	1.000			
Female	24 (28.6)	9 (28.1)	0.978 (0.396–2.417)	0.962		
**Age, N (%)**						
< 65 years	36 (42.9)	13 (40.6)	1.000			
≥ 65 years	48 (57.1)	19 (59.4)	1.096 (0.479–2.507)	0.828		
**Body mass index, N (%)**						
< 22 kg/m^2^	39 (46.4)	21 (65.6)	1.000			
≥ 22 kg/m^2^	45 (53.6)	11 (34.4)	2.203 (0.945–5.134)	0.067		
**Smoking, N (%)**						
None	38 (45.2)	6 (18.8)	1.000		1.000	
Yes	46 (54.8)	26 (81.2)	3.580 (1.335–9.597)	0.011	3.451 (1.240–9.607)	0.018
**Hypertension, N (%)**						
Absence	56 (66.7)	21 (65.6)	1.000			
Presence	28 (33.3)	11 (34.4)	1.048 (0.444–2.473)	0.915		
**Diabetes mellitus, N (%)**						
Absence	73 (86.9)	27 (84.4)	1.000			
Presence	11 (13.1)	5 (15.6)	1.229 (0.391–3.864)	0.724		
**Chemoradiotherapy, N (%)**						
Absence	57 (67.9)	23 (71.9)	1.000			
Presence	27 (32.1)	9 (28.1)	0.826 (0.337–2.025)	0.676		
**Clinical T status, N (%)**						
1 and 2	30 (35.7)	17 (53.1)	1.000			
3 and 4	54 (64.3)	15 (46.9)	0.490 (0.215–1.119)	0.090		
**Clinical N status, N (%)**						
Negative	54 (64.3)	19 (59.4)	1.000			
Positive	30 (35.7)	13 (40.6)	1.232 (0.534–2.838)	0.625		
**Clinical M status, N (%)**						
Negative	78 (92.9)	32 (100.0)	1.000			
Positive	6 (7.1)	0 (0.0)	–	–		
**Final T status, N (%)**						
1 and 2	36 (42.9)	18 (56.2)	1.000			
3 and 4	48 (57.1)	14 (43.8)	0.583 (0.257–1.326)	0.198		
**Final N status, N (%)**						
Negative	59 (70.2)	20 (62.5)	1.000			
Positive	25 (29.8)	12 (37.5)	1.416 (0.602–3.329)	0.425		
**Final M status, N (%)**						
Negative	79 (94.0)	32 (100.0)	1.000			
Positive	5 (6.0)	0 (0.0)	–	–		
**Albumin level, N (%)**						
< 3.5 g/dL	16 (19.0)	4 (12.5)	1.000			
≥ 3.5 g/dL	68 (81.0)	28 (87.5)	1.647 (0.506–5.364)	0.408		
**Total cholesterol level, N (%)**						
< 180 mg/dL	25 (29.8)	13 (40.6)	1.000			
≥ 180 mg/dL	59 (70.2)	19 (59.4)	0.619 (0.265–1.444)	0.267		
**Lymphocyte count, N (%)**						
< 1600 cells/µL	45 (53.6)	16 (50.0)	1.000			
≥ 1600 cells/µL	39 (46.4)	16 (50.0)	1.154 (0.511–2.606)	0.731		
**PNI, N (%)**						
≤ 40	33 (39.3)	13 (40.6)	1.000			
> 40	51 (60.7)	19 (59.4)	0.946 (0.412–2.169)	0.895		
**GNRI, N (%)**						
Normal	60 (71.4)	26 (81.2)	1.000			
Low-severe	24 (28.6)	6 (18.8)	0.577 (0.211–1.578)			
**CONUT score, N (%)**						
Normal	43 (51.2)	17 (53.1)	1.000			
Light-severe	41 (48.8)	15 (46.9)	0.925 (0.409–2.092)			
**Operation time, N (%)**						
< 360 min	32 (38.1)	12 (37.5)	1.000			
≥ 360 min	52 (61.9)	20 (62.5)	1.026 (0.443–2.376)	0.953		
**Blood loss, N (%)**						
< 100 mL	25 (29.8)	14 (43.8)	1.000			
≥ 100 mL	59 (70.2)	18 (56.2)	0.545 (0.235–1.263)	0.157		
**Approach type, N (%)**						
Laparoscopy	57 (67.9)	28 (87.5)	1.000		1.000	
Open	27 (32.1)	4 (12.5)	0.302 (0.096–0.946)	0.040	0.324 (0.099–1.065)	0.063
**Stoma type, N (%)**						
Colostomy	45 (53.6)	8 (25.0)	1.000		1.000	
Ileostomy	39 (46.4)	24 (75.0)	3.462 (1.396–8.581)	0.007	3.287 (1.278–8.458)	0.014
**Stoma position, N (%)**						
Upper abdomen	14 (16.7)	7 (21.9)	1.000			
Lower abdomen	70 (83.3)	25 (78.1)	0.714 (0.259–1.972)	0.516		

*PSDs* peristomal skin disorders, *OR* odds ratio, *CI* confidence interval, *PNI* prognostic nutritional index, *GNRI* geriatric nutritional risk index, *CONUT* Controlling Nutritional Status

Severe PSDs (based on an ABCD-stoma score ≥ 4) were diagnosed in 10 patients (8.7%) a median of 20.5 (range 14.0–37.0) days after surgery. Lymphocyte count (p = 0.037) and CONUT score (p = 0.024) were significantly associated with severe PSDs in the univariate analysis (Table 3). The CONUT score (OR 10.040, 95% CI 1.191–84.651, p = 0.034) was an independent risk factor for severe PSD in the multivariable analysis.

**Table 3:** Risk factors for severe peristomal skin disorders.

	Non-severe PSDs, N = 106	Severe PSDs, N = 10	Univariate analysis		Multivariable analysis	
			OR (95% CI)	p	OR (95% CI)	p
**Sex, N (%)**						
Male	75 (70.8)	8 (80.0)	1.000			
Female	31 (29.2)	2 (20.0)	0.605 (0.122–3.011)	0.539		
**Age, N (%)**						
< 65 years	47 (44.3)	2 (20.0)	1.000			
≥ 65 years	59 (55.7)	8 (80.0)	3.186 (0.646–15.722)	0.155		
**Body mass index, N (%)**						
< 22 kg/m^2^	53 (50.0)	7 (70.0)	1.000			
≥ 22 kg/m^2^	53 (50.0)	3 (30.0)	2.333 (0.572–9.510)	0.237		
**Smoking, N (%)**						
None	43 (40.6)	1 (10.0)	1.000		1.000	
Yes	63 (59.4)	9 (90.0)	6.143 (0.751–50.266)	0.091	5.247 (0.608–45.318)	0.132
**Hypertension, N (%)**						
Absence	43 (40.6)	1 (10.0)	1.000		1.00	
Presence	63 (59.4)	9 (90.0)	3.318 (0.877–12.550)	0.077	3.547 (0.862–14.596)	0.079
**Diabetes mellitus, N (%)**						
Absence	92 (86.8)	8 (80.0)	1.000			
Presence	14 (13.2)	2 (20.0)	1.643 (0.316–8.540)	0.555		
**Chemoradiotherapy, N (%)**						
Absence	75 (70.8)	5 (50.0)	1.000			
Presence	31 (29.2)	5 (50.0)	2.419 (0.654–8.952)	0.186		
**Clinical T status, N (%)**						
1 and 2	45 (42.5)	2 (20.0)	1.000			
3 and 4	61 (57.5)	8 (80.0)	2.951 (0.598–14.566)	0.184		
**Clinical N status, N (%)**						
Negative	66 (62.3)	7 (70.0)	1.000			
Positive	40 (37.7)	3 (30.0)	0.707 (0.173–2.892)	0.630		
**Clinical M status, N (%)**						
Negative	100 (94.3)	10 (100.0)	1.000			
Positive	6 (5.7)	0 (0.0)	–	–		
**Final T status, N (%)**						
1 and 2	51 (48.1)	3 (30.0)	1.000			
3 and 4	55 (51.9)	7 (70.0)	2.164 (0.531–8.819)	0.282		
**Final N status, N (%)**						
Negative	71 (67.0)	8 (80.0)	1.000			
Positive	35 (33.0)	2 (20.0)	0.507 (0.102–2.516)	0.406		
**Final M status, N (%)**						
Negative	101 (95.3)	10 (100.0)	1.000			
Positive	5 (4.7)	0 (0.0)	–	–		
**Albumin level, N (%)**						
< 3.5 g/dL	17 (!6.0)	3 (30.0)	1.000			
≥ 3.5 g/dL	89 (84.0)	7 (70.0)	0.446 (0.105–1.897)	0.274		
**Total cholesterol level, N (%)**						
< 180 mg/dL	33 (31.1)	5 (50.0)	1.000			
≥ 180 mg/dL	73 (68.9)	5 (50.0)	0.452 (0.122–1.669)	0.233		
**Lymphocyte count, N (%)**						
< 1600 cells/µL	52 (49.1)	9 (90.0)	1.000			
≥ 1600 cells/µL	54 (50.9)	1 (10.0)	0.107 (0.013–0.874)	0.037		
**PNI, N (%)**						
≤ 40	43 (40.6)	3 (30.0)	1.000			
> 40	63 (59.4)	7 (70.0)	1.593 (0.390–6.503)	0.517		
**GNRI, N (%)**						
Normal	79 (74.5)	7 (70.0)	1.000			
Low-severe	27 (25.5)	3 (30.0)	1.254 (0.303–5.195)	0.755		
**CONUT score, N (%)**						
Normal	59 (55.7)	1 (10.0)	1.000		1.000	
Light-severe	47 (44.3)	9 (90.0)	11.298 (1.382–92.373)	0.024	10.040 (1.191–84.615)	0.034
**Operation time, N (%)**						
< 360 min	39 (36.8)	5 (50.0)	1.000			
≥ 360 min	67 (63.2)	5 (50.0)	0.582 (0.158–2.138)	0.582		
**Blood loss, N (%)**						
< 100 mL	37 (34.9)	2 (20.0)	1.000			
≥ 100 mL	69 (65.1)	8 (80.0)	2.145 (0.433–10.625)	0.350		
**Approach type, N (%)**						
Laparoscopy	78 (73.6)	7 (70.0)	1.000			
Open	28 (26.4)	3 (30.0)	1.194 (0.289–4.938)	0.807		
**Stoma type, N (%)**						
Colostomy	49 (46.2)	4 (40.0)	1.000			
Ileostomy	57 (53.8)	6 (60.0)	1.289 (0.344–4.834)	0.706		
**Stoma position, N (%)**						
Upper abdomen	87 (82.1)	2 (20.0)	1.000			
Lower abdomen	19 (17.9)	8 (80.0)	0.874 (0.172–4.445)	0.871		

*PSDs* peristomal skin disorders, *OR* odds ratio, *CI* confidence interval, *PNI* prognostic nutritional index, *GNRI* geriatric nutritional risk index, *CONUT* Controlling Nutritional Status.

**Figure 2:**
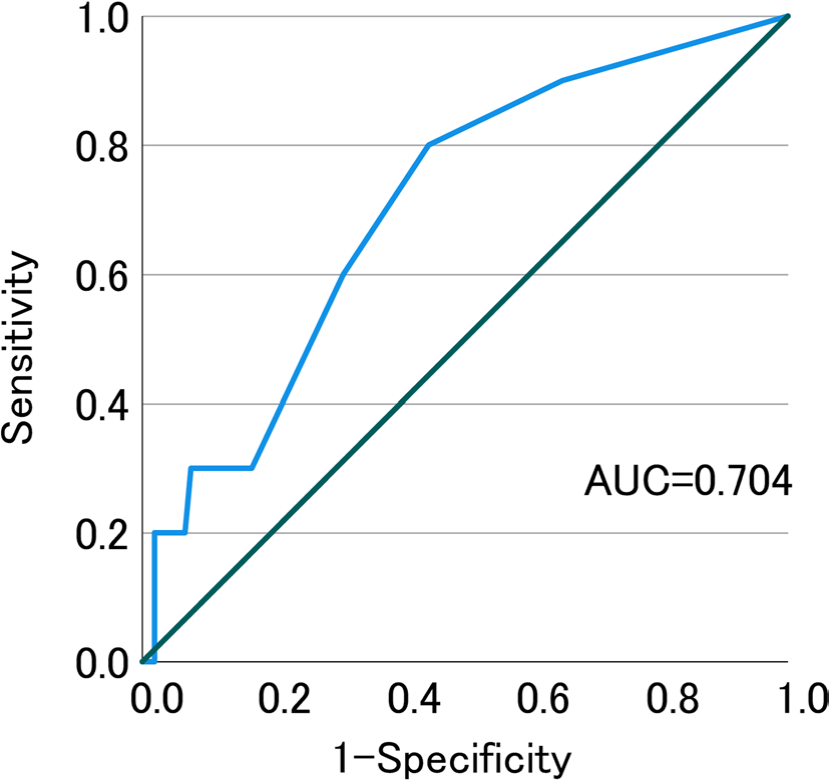
Prediction of the development of severe PSDs according to the CONUT score. *AUC* area under the curve.

The receiver-operating characteristic curve (ROC) was used to determine the nutritional disorder based on CONUT score that optimally predicted the development of severe PSDs. The area under the curve (AUC) for CONUT scores was 0.704 (95% CI 0.544–0.865). A cutoff value of 1.5 was selected, which maximized specificity and sensitivity (80.0% and 55.7%, respectively), for the prediction of the development of severe PSDs based on CONUT score (Fig. 2).

## Discussion

The results of this study showed that severe PSDs were strongly associated with preoperative nutritional disorders, as evaluated by the CONUT score. Furthermore, severe PSDs occurred in patients with stoma and preoperative nutritional disorders, regardless of stoma type. Patients with ileostomies were significantly more likely to develop PSDs than patients with colostomies, supporting the results of previous studies. Almost all of ileostomy-associated PSDs were non-severe. These findings highlight the importance of stoma care, and the need for clinicians to be especially vigilant in the prevention of PSDs in ileostomy patients and patients with poor preoperative nutritional status. Additionally, severe PSDs may be prevented by the appropriate management of preoperative nutritional disorders.

Preoperative nutritional disorders evaluated via the CONUT score were associated with severe PSDs. Nutritional deficiencies are a well-established cause of skin disorders^14^. Although the skin functions normally when adequate nutrition is provided, a deficiency of essential fatty acids increases epidermal permeability and transepidermal water loss^15^. As a result, the skin becomes vulnerable to mechanical stimulation, such as that which occurs during stoma appliance replacement and drainage of feces; the changing of stoma appliances requires a pulling force to remove the pouch system, which is attached to the skin with an adhesive paste. This leads to the removal of the stratum corneum^16^, and severe PSDs can result from the separation of the epidermis from the dermis, particularly when skin function is impaired due to a poor nutritional status. While initial changes may only comprise peristomal skin erythema, prolonged mechanical damage can result in erosion, ulceration, and blistering^17^. Additionally, nutritional disorders can hinder the recovery of skin damage and increase the risk of severe PSDs. This may account for our observation that nutritional disorders (as evaluated by the CONUT score) were only significantly associated with severe PSDs, and not all PSDs. Our results show that the CONUT score is a useful and cost-effective method for the objective and comprehensive evaluation the nutritional status of stoma patients. Furthermore, it may be used to predict the severity of postoperative PSD.

In preoperative nutritional evaluations, only CONUT scores were associated with severe PSDs. The lipid bilayer of the stratum corneum has an important role in the barrier function of the skin, and requires ceramides, which are naturally occurring lipids^18^. Ceramides are essential for the prevention of transepidermal water loss, as they fuse corneocytes in the stratum corneum, thus forming a protective layer. In contrast to other nutritional indices such as PNI and GNRI, the CONUT score includes the cholesterol level as a lipid indicator. Therefore, only preoperative CONUT scores, including the indicator of a lipid involved in an important role in the barrier function of the skin, were associated with severe PSDs.

Ileostomy was associated with both mild and severe PSDs, supporting the results of previous studies^19,20^. The majority of these PSDs were not severe. Ileostomy commonly results in PSDs, as the liquid removed from the stoma contains a highly active and caustic soft stool, which has digestive and proteolytic enzymes; furthermore, frequent changes in stoma appliance are required^21^. Nevertheless, severe PSDs were documented in patients with both ileostomies and colostomies. PSDs in cases involving ileostomy generally improve without the development of severe PSDs in patients with a normal nutritional status, as all skin functions are intact. However, increased vigilance is required to monitor the development of severe PSDs in patients with preoperative nutritional disorders, regardless of the stoma type. Preventive management should not only include the selection of appropriate appliances and the use of ceramide-containing skin protectants to minimize damage to the skin^3^, but also interventions to improve preoperative nutritional status.

Smoking was associated with all PSDs. Smoking has been shown to adversely affect various organ systems, including the skin. Various harmful substances contained in tobacco, such as nicotine, cause an increase in microvascular occlusion and tissue ischemia of skin^22^, and a decrease in collagen synthesis and fibroblast proliferation^23^. As a result, smoking induces delayed wound healing, and an increased risk of infections. Although smoking was associated with all PSDs, all patients had quit smoking prior to surgery. This may have prevented the PSDs from becoming severe. This result reaffirms the importance of not smoking.

The study had several limitations. First, it had a single-center retrospective design, and the sample size was relatively small, especially the number of severe PSDs. Further studies with larger sample sizes are required to clarify the association between PSDs and nutritional status. Second, information on the number of stoma leaks, appliance replacements, and stoma height were not available in the medical records; these factors have been previously associated with PSDs. Third, we could not directly evaluate the relationship between the preoperative skin condition and nutrition, as the former was not adequately evaluated and documented in the clinical records. Nonetheless, the results of this study indicate that the CONUT score can be used to facilitate the identification of patients at high risk of severe PSDs, who require nutritional intervention.

In conclusion, severe PSDs were strongly associated with preoperative nutritional disorders, as evaluated by the CONUT score. Furthermore, severe PSDs occurred in stoma patients with preoperative nutritional disorders, regardless of stoma type. While PSDs were significantly associated with ileostomy, the majority were not severe. Our findings highlight the importance of stoma care, and the need for clinicians to be especially vigilant in the prevention of PSDs in patients with ileostomies or poor preoperative nutritional status.

## References

[1] Nybaek H, Knudsen DB, Laursen TN, Karlsmark T, Jemec GB (2010). Quality of life assessment among patients with peristomal skin disease. Eur. J. Gastroenterol. Hepatol..

[2] Chambers SK (2012). A five-year prospective study of quality of life after colorectal cancer. Qual. Life Res..

[3] Colwell JC, Pittman J, Raizman R, Salvadalena G (2018). A randomized controlled trial determining variances in ostomy skin conditions and the economic impact (ADVOCATE Trial). J. Wound Ostomy Continence Nurs..

[4] Taneja C (2017). Clinical and economic burden of peristomal skin complications in patients with recent ostomies. J. Wound Ostomy Continence Nurs..

[5] Shiraishi T (2020). Risk factors for the incidence and severity of peristomal skin disorders defined using two scoring systems. Surg. Today.

[6] Finner AM (2013). Nutrition and hair: Deficiencies and supplements. Dermatol. Clin..

[7] Meyer NA, Muller MJ, Herndon DN (1994). Nutrient support of the healing wound. New Horiz..

[8] Buzby GP, Mullen JL, Matthews DC, Hobbs CL, Rosato EF (1980). Prognostic nutritional index in gastrointestinal surgery. Am. J. Surg..

[9] Sun G (2019). Impact of the preoperative prognostic nutritional index on postoperative and survival outcomes in colorectal cancer patients who underwent primary tumor resection: A systematic review and meta-analysis. Int. J. Colorectal Dis..

[10] Bouillanne O (2005). Geriatric Nutritional Risk Index: A new index for evaluating at-risk elderly medical patients. Am. J. Clin. Nutr..

[11] de Ulíbarri JI (2005). CONUT: A tool for controlling nutritional status. First validation in a hospital population. Nutr. Hosp..

[12] Tokunaga R (2017). CONUT: A novel independent predictive score for colorectal cancer patients undergoing potentially curative resection. Int. J. Colorectal Dis..

[13] Hayama T (2020). The pretreatment Controlling Nutritional Status (CONUT) score is an independent prognostic factor in patients undergoing resection for colorectal cancer [sci. rep.:13239]. Sci. Rep..

[14] Basavaraj KH, Seemanthini C, Rashmi R (2010). Diet in dermatology: Present perspectives. Indian J. Dermatol..

[15] Boelsma E, Hendriks HF, Roza L (2001). Nutritional skin care: Health effects of micronutrients and fatty acids. Am. J. Clin. Nutr..

[16] Dykes PJ, Heggie R, Hill SA (2001). Effects of adhesive dressings on the stratum corneum of the skin. J. Wound Care.

[17] Brett DW (2006). Impact on pain control, epidermal stripping, leakage of wound fluid, ease of use, pressure reduction, and cost-effectiveness. J. Wound Ostomy Continence Nurs..

[18] Coderch L, López O, de la Maza A, Parra JL (2003). Ceramides and skin function. Am. J. Clin. Dermatol..

[19] Robertson I (2005). Prospective analysis of stoma-related complications. Colorectal Dis..

[20] Ratliff CR, Donovan AM (2001). Frequency of peristomal complications. Ostomy Wound Manag..

[21] Almutairi D, LeBlanc K, Alavi A (2018). Peristomal skin complications: What dermatologists need to know. Int. J. Dermatol..

[22] Wennmalm A, Alster P (1983). Nicotine inhibits vascular prostacyclin but not platelet thromboxane formation. Gen. Pharmacol..

[23] Su Y, Cao W, Han Z, Block ER (2004). Cigarette smoke extract inhibits angiogenesis of pulmonary artery endothelial cells: The role of calpain. Am. J. Physiol. Lung Cell. Mol. Physiol..

